# Why does my son have a genetic disease?

**DOI:** 10.1186/1824-7288-41-S2-A18

**Published:** 2015-09-30

**Authors:** Lorella Congiunti

**Affiliations:** 1 Faculty of Philosophy, Pontifical Urbaniana University, Vatican City 00120, Italy; 2 Pontifical Urbaniana University, Vatican City 00120, Italy

## 

The question “why” is a typical question of human beings, who not only know the world and react to it, but also wish to understand its meaning.

From the classical reflection of Aristotle which was conducted in physics as well as in metaphysics on the fourfold dimension of causality, important observations are still deduced. The cause is always material, formal, efficient, final.

Every time, in fact, one asks the question “why”, the person is making a complex question which contains others: why is he/she so, what is the reason, who is the person that has done this, what is the aim.

In front of the genetic disease of one's son, the question “why” reveals one's urgency and necessity, and at the same time imposes the awareness that it is necessary to avoid silence as well as non-response, or the arrogance of a definite answer.

The philosophic reflection proposes research outlines on the causes, on the subjects that can and should investigate them, on the relationship between fields of knowledge, above all in relation to notions connected to “life”[1].

Is life only a medical notion? Is it only philosophical? Or only religious? And should disease be studied only from a medical perspective? Or only philosophical? Or only religious?

The most efficient answers come from the composition of knowledge; only this is able to hold together the specificity of knowledge and the complexity of reality [2].

## References

[B1] Congiunti L La natura umana tra scienza e filosofia Nuova Civiltà delle Macchine 2012 112 127

[B2] Congiunti L Lineamenti di filosofia della natura 2010 Urbaniana University Press 1 331

